# Direct Vasocontractile Activities of Bupivacaine Enantiomers on the Isolated Rat Thoracic Aorta

**DOI:** 10.1155/2010/820186

**Published:** 2010-10-26

**Authors:** Mai Mukozawa, Ko Takakura, Maki Mizogami

**Affiliations:** Department of Anesthesiology, Asahi University, 1851 Hozumi, Gifu 5010296, Japan

## Abstract

*Background*. *In vitro* studies with isolated arteries have shown direct vasoactivity of racemic bupivacaine. However, there is little information on the direct vasoactivities of bupivacaine enantiomers, S(−)- and R(+)-bupivacaine. *Methods*. We performed functional examinations using isolated intact thoracic aortic rings from male Wistar rats. Changes in ring tension produced by S(−)-, R(+)-, or racemic bupivacaine were measured in Krebs solution. *Results*. S(−)-bupivacaine produced the strongest contraction of the three agents. R(+)-bupivacaine showed limited vasoconstriction. The effects of racemic bupivacaine were located between these two. *Conclusion*. Each bupivacaine enantiomer showed specific vasocontractile activity, which affects the activity of racemic bupivacaine.

## 1. Introduction

The local anesthetic bupivacaine is a racemic mixture of S(−)- and R(+)-enantiomers. Racemic bupivacaine has biphasic vasoactivities, namely, vasoconstriction at a low concentration and vasodilatation at a high concentration [1]. Since 1976, when Aps and Reynolds showed this vasoactivity in a double-blind trial with forearm skin color changes of 31 volunteers [1], these vasoactivities have been further demonstrated using various *in vivo* methods with various animals or humans, such as television microscopy in rat cremaster muscle microvasculatures [2], intravital microscopy through a spinal window in dog pial vasculatures [3], laser Doppler imaging in human skin [4], as well as other techniques. Although inhibition of sympathetic nerves innervating arteries by racemic bupivacaine could not be ignored in *in vivo* studies, some *in vitro* studies with isolated preparations from human umbilical arteries [5–7], rat uterine arteries [8], and human uterine arteries [9] have confirmed that the vasoactivities are produced by direct actions of racemic bupivacaine itself on the arteries. 

S(−)-bupivacaine was developed as an alternative long-acting local anesthetic with a clinical profile similar to that of racemic bupivacaine but with a lower potential for producing systemic toxicity [10]. S(−)-bupivacaine also has biphasic vasoactivities similar to those of racemic bupivacaine, which has been shown in* in vivo* studies [4, 11–13]. Furthermore, some of the *in vivo* studies have shown that R(+)-bupivacaine produced a dose-dependent vasodilatation [11, 13]. However, there is little information on the direct vasoactivities of S(−)- and R(+)-bupivacaine based on *in vitro* study. In this study, we investigated the vasocontractile activities of these agents using isolated rat aorta.

## 2. Methods

### 2.1. Animals

The experimental protocol was approved by the institutional animal care committee of Asahi University. Male Wistar rats weighing 240–280 g were used.

### 2.2. Functional Experiments

Rats were killed by decapitation under sevoflurane anesthesia, and the thoracic aorta was isolated and removed [14]. The thoracic aorta was placed in Krebs-Henseleit solution (mM; NaCl 118, KCl 4.7, NaHCO_3_ 25, KH_2_PO_4_ 1.2, MgSO_4_ 1.2, CaCl_2_ 2.5, and glucose 10; pH 7.4). Aortic rings were carefully prepared under a dissecting microscope, and then each intact ring was carefully pulled by wires in an organ chamber containing 5 mL Krebs-Henseleit solution bubbled with 95% O_2_ and 5% CO_2_ at 37°C. After a resting tension of 1.0 g was applied during one-hour equilibration period, changes in the tension were recorded isometrically when S(−)-bupivacaine, R(+)-bupivacaine, or racemic bupivacaine was cumulatively applied. Contractions were expressed as mg contractile tension.

### 2.3. Chemicals

S(−)-bupivacaine and R(+)-bupivacaine were generously donated by Maruishi Pharmaceutical (Osaka, Japan), and pseudoracemic bupivacaine was prepared by mixing with S(−)-bupivacaine and R(+)-bupivacaine at a ratio of 1 : 1 [15].

### 2.4. Statistical Analysis

The results are expressed as mean ± SD. The maximum response (E_max_\) and the concentration producing a half-maximal response (EC_50_) were determined by Finley's probit analysis. Significance of differences was analyzed by Kruskal-Wallis test and Scheffé method as a post hoc comparison for multiple comparisons at a significance level of  0.05.

## 3. Results

**Figure 1:**
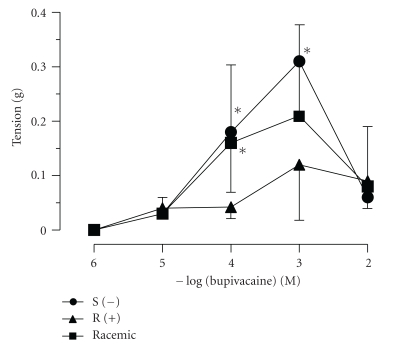
Changes in vascular tension provoked by S(−)-, R(+)-, and racemic bupivacaines. *:  *P* < .05 versus R(+)-bupivacaine. *n* = 8.

S(−)-bupivacaine, R(+)-bupivacaine, and racemic bupivacaine produced a biphasic response in the aortic rings, namely, concentration-dependent contraction from 10 *μ*M to 1 mM and relaxation at higher concentrations (*n* = 8, Figure 1.) The vasocontractile responses produced by S(−)-bupivacaine and racemic bupivacaine were significantly stronger than those by R(+)-bupivacaine. Furthermore, there were significant differences among E_max_ or EC_50_ of each bupivacaine (Table 1).

## 4. Discussion

As there has been only limited study of the direct vasoactivities of two bupivacaine enantiomers, S(−)- and R(+)-bupivacaine, we compared the vasocontractile effects of these two agents with that of racemic bupivacaine in this study. S(−)-bupivacaine showed the strongest E_max_ of the three agents, while R(+)-bupivacaine showed limited vasoconstriction. Although racemic bupivacaine produced as much E_max_ as S(−)-bupivacaine statistically (Table 1), the activity level of racemic bupivacaine was located between those of S(−)-bupivacaine and R(+)-bupivacaine graphically (Figure 1). R(+)-bupivacaine, which produced small vasoconstriction even at high concentration, may interfere with vasoconstriction by S(−)-bupivacaine in racemic bupivacaine, which consists of the two enantiomers. Several *in vitro* studies have been done on racemic bupivacaine's direct vasoactivities. It has been reported that racemic bupivacaine contracted isolated rat uterine arteries [8], human uterine arteries [9], human umbilical arteries [5–7], and veins [6] in various degrees. Different vasocontractile activities of the enantiomers among the vessels might be, at least, a cause of the variety.

S(−)-bupivacaine produced the strongest vasoconstriction of the three bupivacaines in our study. Considering that the clinical use of S(−)-bupivacaine is increasing because of its lower toxicity [10], it is important to note that it may produce greater vasoconstriction than racemic bupivacaine does. Bupivacaine administered for epidural anesthesia raises the intrathecal and plasma concentration [16] and might contract several important vessels, including the pial, epidural, uterine, umbilical arteries, as well as others, with subsequent decrease of blood flow.

## Figures and Tables

**Table 1:** EC_50_ values and maximum contraction by bupivacaines.

Bupivacaine	EC_50_(×10^−5^)	E_max_ (g)
S (−)	7.3 ± 2.9	0.31 ± 0.06
R (+)	4.1 ± 4.0*	0.12 ± 0.10*
Racemic	4.1 ± 1.6*	0.21 ± 0.10

*：*P* < .05 versus S(−). *n* = 8.
